# Divergent Thyroid Hormone Levels in Plasma and Left Ventricle of the Heart in Compensated and Decompensated Cardiac Hypertrophy Induced by Chronic Adrenergic Stimulation in Mice

**DOI:** 10.3390/metabo13020308

**Published:** 2023-02-20

**Authors:** Warner Simonides, Alice Tijsma, Anita Boelen, Rutchanna Jongejan, Yolanda de Rijke, Robin Peeters, Monica Dentice, Domenico Salvatore, Alice Muller

**Affiliations:** 1 Department of Physiology, Amsterdam UMC, Vrije Universiteit, De Boelelaan 1118, 1081 HV Amsterdam, The Netherlands; 2 Amsterdam Cardiovascular Sciences, Heart Failure & Arrhythmias, 1081 HZ Amsterdam, The Netherlands; 3 Endocrine Laboratory, Department of Clinical Chemistry, Amsterdam UMC, University of Amsterdam, Meibergdreef 9, 1105 AZ Amsterdam, The Netherlands; 4 Department of Clinical Chemistry, Erasmus MC University Medical Center, Dr. Molewaterplein 40, 3000 CA Rotterdam, The Netherlands; 5 Department of Internal Medicine, Erasmus MC University Medical Center, Dr. Molewaterplein 40, 3000 CA Rotterdam, The Netherlands; 6 Department of Clinical Medicine and Surgery, University of Naples “Federico II”, Via Pansini 5, 80131 Naples, Italy; 7 Department of Public Health, University of Naples “Federico II”, Via Pansini 5, 80131 Naples, Italy

**Keywords:** thyroid hormone, deiodinase type 3, adrenergic agonist, cardiac hypertrophy, mouse

## Abstract

Chronic hemodynamic overload of the heart induces ventricular hypertrophy that may be either compensatory or progress to decompensation and heart failure. The gradual impairment of ventricular function is, at least in part, the result of a reduction of cardiac thyroid-hormone (TH) action. Here, we examined the proposed roles of increased cardiac expression of the TH-inactivating enzyme deiodinase type 3 (D3) and reduced plasma TH levels in diminishing cardiac TH levels. Using minipumps, mice were infused for one and two weeks with isoproterenol (ISO) alone or in combination with phenylephrine (PE). Remodeling of the heart induced by these adrenergic agonists was assessed by echocardiography. Left ventricular (LV) tissue and plasma TH levels (T4 and T3) were determined using liquid chromatography-tandem mass spectrometry. LV D3 activity was determined by conversion of radiolabeled substrate and quantification following HPLC. The results show that ISO induced compensated LV hypertrophy with maintained cardiac output. Plasma levels of T4 and T3 remained normal, but LV hormone levels were reduced by approximately 30% after two weeks, while LV D3 activity was not significantly increased. ISO + PE induced decompensated LV hypertrophy with diminished cardiac output. Plasma levels of T4 and T3 were substantially reduced after one and two weeks, together with a more than 50% reduction of hormone levels in the LV. D3 activity was increased after one week and returned to control levels after two weeks. These data show for the first time that relative to controls, decompensated LV hypertrophy with diminished cardiac output is associated with a greater reduction of cardiac TH levels than compensated hypertrophy with maintained cardiac output. LV D3 activity is unlikely to account for these reductions after two weeks in either condition. Whereas the mechanism of the mild reduction in compensated hypertrophy is unclear, changes in systemic TH homeostasis appear to determine the marked drop in LV TH levels and associated impairment of ventricular function in decompensated hypertrophy.

## 1. Introduction

The heart has the capacity to adapt to mild increases in hemodynamic load, e.g., endurance training and pregnancy, by cardiomyocyte growth and concomitant ventricular hypertrophy, which normalizes wall stress and maintains cardiac output and contractile efficiency [1]. This capacity for physiological compensatory hypertrophy may reach its limits when a chronic pressure and/or volume overload is caused by hypertension, aortic stenosis, valvular disease, cardiac ischemia or loss of functional tissue due to myocardial infarction (MI). An initially compensatory phase then progresses to a condition of chronic heart failure, characterized by progressive contractile dysfunction, cell death, fibrosis, inflammation and capillary rarefaction, ultimately culminating in decompensation and end-stage heart failure.

Numerous factors are involved in the mechanical, hormonal and neuro-hormonal signal-transduction pathways that drive this form of pathological ventricular remodeling [1,2,3,4]. Virtually every aspect of cardiac contractility, energy metabolism and myocyte shape is to some degree regulated by the thyroid hormone (TH) through both direct and indirect genomic mechanisms [5,6,7,8]. The involvement of reduced TH signaling in pathological ventricular remodeling is suggested by similar changes in critical cardiac gene expression in both hypothyroidism and heart failure, and by the reduction of plasma TH levels that is seen in heart failure and other critical illnesses [9,10,11]. In addition, induction of the TH-inactivating type 3 deiodinase (D3) in cardiomyocytes early in the process of ventricular remodeling has been shown in various models [12,13,14,15,16,17,18]. D3 converts the pro-hormone T4 (3,5,3′,5′-tetraiodothyronine) and the physiologically more active form T3 (3,5,3′-triiodothyronine) to the inactive metabolites reverse-T3 (rT3) (3,3′,5′-triiodothyronine) and T2 (3,3′-diiodothyronine), respectively [19,20]. D3 activity is high in fetal tissues but virtually absent in most adult tissues, including the heart [20].

The induction of D3 in the remodeling ventricle is thought to be part of the re-expression of the gene program driving fetal growth [21], and the associated deiodinating activity is hypothesized to play a role in the reduction of TH signaling in hypertrophic cardiomyocytes. In fact, increased D3 activity is correlated with a reduction of cardiac TH-dependent transcription and TH levels in right-ventricular (RV) hypertrophy and failure induced by pulmonary arterial hypertension in rats [12,13], as well as in left-ventricular (LV) hypertrophy following MI in mice [14].

In the present study, we used the established method of chronic adrenergic stimulation to induce compensated and decompensated LV hypertrophy [17,22,23,24,25,26,27,28,29,30]. The aim was to provide, for the first time, a comprehensive analysis of the development of compensated and decompensated LV hypertrophy in a model of chronic hemodynamic overload, determining cardiac morphology and function, systemic and LV tissue TH levels, as well as LV D3 activity. We used a mouse line in which the expression of active cardiac D3 protein can be conditionally reduced, which in future studies will allow for the assessment of the relevance of D3 activity for pathological ventricular hypertrophy.

## 2. Results

It should be noted that D3 expression and enzyme activity are normal in the cD3KO-CS mice used in this study since these mice were not induced to express inactive cardiac D3 protein (see Materials and Methods).

### 2.1. Isoproterenol Induces Compensated Left-Ventricular Hypertrophy in cD3KO-CS Mice

#### 2.1.1. Cardiac Parameters of ISO-Infused cD3KO-CS Mice

**Figure 1:**
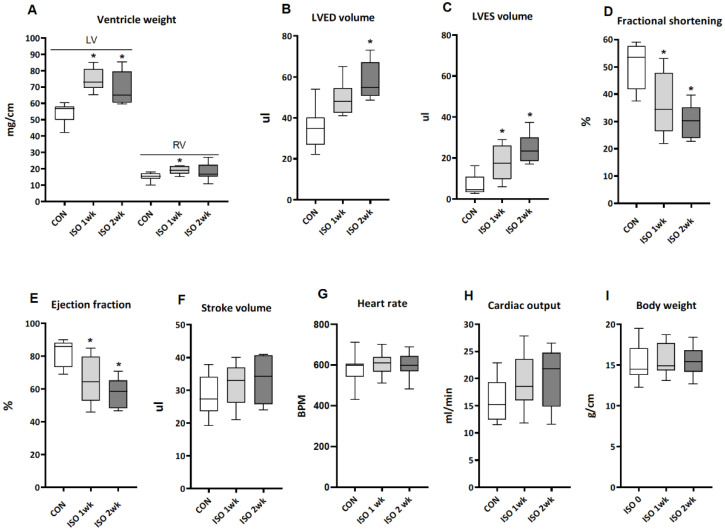
The effect of one and two weeks of ISO infusion on cardiac parameters and body weight. (**A**) Ventricle weight was normalized to tibia length. Data in panels (**B**–**H**) were obtained by echocardiography. (**B**) LVED: left ventricular end-diastolic; (**C**) LVES: left ventricular end-systolic. Data are presented as median and quartiles with whiskers indicating minimum and maximum values. (**A**–**H**): *n* = 6–10 for all groups; (**I**): *n* = 16 for 0, 1 week and *n* = 8 for 2 week. Two-way ANOVA followed by unprotected Fisher’s LSD test was used to determine significance of differences (**A**–**H**). Paired Student *t*-tests were used in the case of body weight changes (**I**). *: *p* < 0.05 vs. CON.

The infusion of ISO for one and two weeks resulted in a 40% increase in LV weight normalized to tibia length after one week, with no further increase in the second week. RV weight increased by approximately 20% only at week one (Figure 1A). LV end diastolic (LVED) and end systolic (LVES) volumes increased substantially (Figure 1B,C). This was associated with an approximate 45% decrease in fractional shortening and a 25% lower ejection fraction (Figure 1D,E). Heart rate was not affected, and stroke volume and cardiac output were maintained as a result of the increase in volume, in spite of the reduction of the ejection fraction (Figure 1F–H).

These results show that an infusion of ISO for two weeks resulted in dilated LV hypertrophy with sufficiently preserved contractility to maintain normal cardiac output. Body weight was not affected by this compensated form of cardiac hypertrophy (Figure 1I).

#### 2.1.2. Thyroid Hormone Levels and Type 3 Deiodinase Activity in ISO-Infused cD3KO-CS Mice

**Figure 2:**
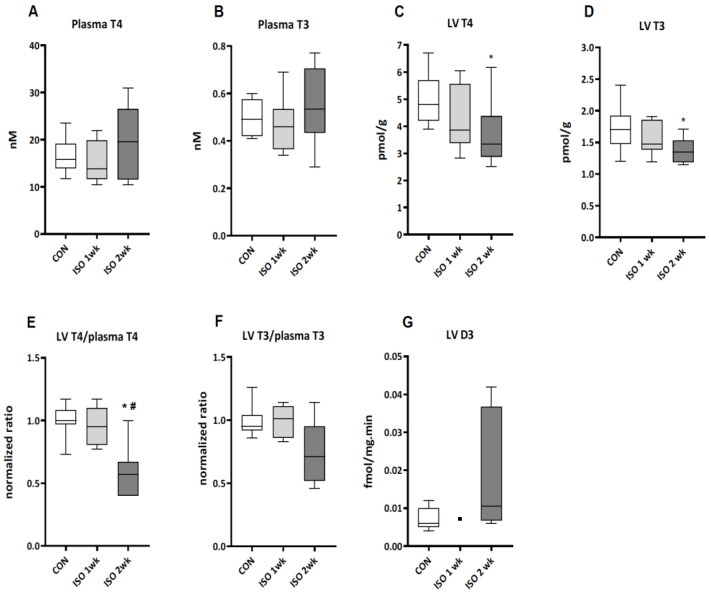
The effect of one and two weeks of ISO infusion on plasma and LV thyroid hormone levels and LV D3 activity. Plasma concentrations (**A**,**B**) and LV tissue content of T4 and T3 (**C**,**D**) were determined by tandem mass spectrometry. Tissue/plasma ratios (**E**,**F**) were normalized to the respective average control value. LV D3 activity (**G**) was determined in tissue homogenates by conversion of radiolabeled T4 followed by HPLC and quantification of metabolites. Data are presented as median and quartiles with whiskers indicating minimum and maximum values. (**A**–**F**): *n* = 8–10 for all groups; (**G**): CON: *n* = 5; ISO 1 week: *n* = 1; ISO 2 week: *n*= 6. Two-way ANOVA followed by unprotected Fisher’s LSD test was used to determine significance of differences. *: *p* < 0.05 vs. CON, #: *p* < 0.05 vs. 1 week.

Plasma levels of T4 and T3 after one and two weeks of ISO infusion were not different from controls (Figure 2A,B). However, the T4 and T3 content of LV tissue was significantly reduced by approximately 30% and 25%, respectively, after two weeks (Figure 2C,D). The average LV tissue/plasma ratio of T4 was about 40% lower versus controls, whereas a borderline significant 30% reduction was seen for T3 (Figure 2E,F). These data indicate a tissue-specific change in TH handling at two weeks. Average LV D3 activity was somewhat increased at this time point, but not significantly different from controls (Figure 2G). Moreover, D3 activities did not show any correlation with tissue T4 or T3 levels in these samples (data not shown).

No difference was found for T3 and T4 plasma levels between control, ISO at week one and ISO at week two, which is in line with compensated LV hypertrophy and preserved cardiac function in these animals. Unexpectedly, the reduced tissue levels of TH in the hypertrophic LV after two weeks cannot be accounted for by the marginal LV D3 activities at week two.

### 2.2. Isoproterenol + Phenylephrine Induces Decompensated Left-Ventricular Hypertrophy in cD3KO-CS Mice

#### 2.2.1. Cardiac Parameters of ISO + PE-Infused cD3KO-CS Mice

**Figure 3:**
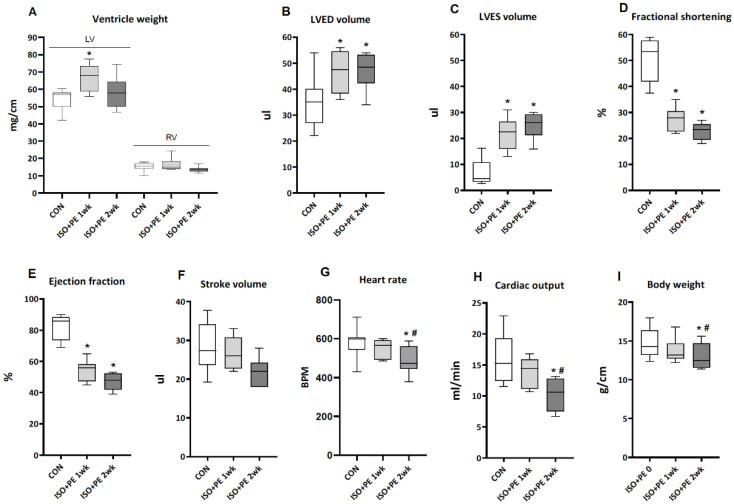
The effect of one and two weeks of ISO + PE infusion on cardiac parameters and body weight. (**A**) Ventricle weight was normalized to tibia length. Data in panels (**B**–**H**) were obtained by echocardiography. (**B**) LVED: left ventricular end-diastolic; (**C**) LVES: left ventricular end-systolic. Data are presented as median and quartiles with whiskers indicating minimum and maximum values. (**A**–**H**): *n* = 6–10 for all groups; (**I**): *n* = 14 for 0, 1 week and *n* = 8 for 2 week. Two-way ANOVA followed by unprotected Fisher’s LSD test was used to determine significance of differences (**A**–**H**). Paired Student *t*-tests were used in the case of body weight changes (**I**). *: *p* < 0.05 vs. CON, #: *p* < 0.05 vs. 1 week.

The infusion of ISO + PE for one and two weeks resulted in an approximate 25% increase in LV weight normalized to tibia length after one week, returning to control levels after two weeks. RV weight was not affected (Figure 3A). LVED and LVES volumes were increased at week one with no further change at week two (Figure 3B,C). This was associated with an approximate 55% decrease in fractional shortening and a 45% lower ejection fraction (Figure 3D,E). In contrast to the ISO data, cardiac output at two weeks decreased significantly by about 30% when compared with those of control and two-week ISO + PE groups (Figure 3H). This was the result of the greater reduction of the ejection fraction at week two and a borderline significant 15% decrease of the stroke volume, together with a marked drop in heart rate at this time point (Figure 3F,G).

Therefore, the infusion of ISO + PE induced dilated LV hypertrophy with impaired contractility and substantially compromised cardiac function. This condition of decompensation and severe illness resulted in significant weight loss over the course of two weeks (Figure 3I).

#### 2.2.2. Thyroid Hormone Levels and Type 3 Deiodinase Activity in ISO + PE-Infused cD3KO-CS Mice

**Figure 4:**
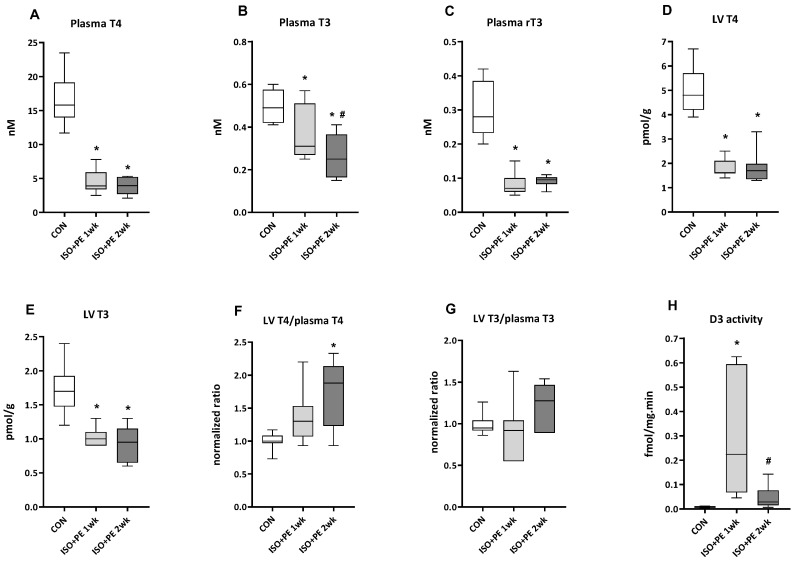
The effect of one and two weeks of ISO + PE infusion on plasma and LV thyroid hormone levels and LV D3 activity. Plasma concentrations (**A**–**C**) of T4, T3 and rT3 and (**C**,**D**) LV tissue content of T4 and T3 were determined by tandem mass spectrometry. Tissue/plasma ratios (**F**,**G**) were normalized to the respective mean control value. LV D3 activity (**H**) was determined in tissue homogenates by conversion of radiolabeled T3 followed by HPLC and quantification of T2. Data are presented as median and quartiles with whiskers indicating minimum and maximum values. (**A**–**G**): *n* = 6–10 for all groups; (**H**): CON: *n* = 5; ISO + PE 1 week: *n* = 7; ISO + PE 2 week: *n*= 8. Two-way ANOVA followed by unprotected Fisher’s LSD test was used to determine significance of differences. *: *p* < 0.05 vs. CON, #: *p* < 0.05 vs. 1 week.

Plasma levels of T4 and T3 showed marked reductions already after one week of ISO + PE infusion (Figure 4A,B). The level of T3 was reduced by approximately 40% after one week compared to control levels, with a further significant reduction to 50% after two weeks. Mean T4 levels decreased by 75% in the first week with no further decrease in the second week. Peripheral conversion of T4 is not suggested to play a role in this decrease since the levels of rT3 decreased in parallel with T4 (Figure 4C). The T4 and T3 content of LV tissue was reduced by about 65% and 40%, respectively, after one week, with no further change after two weeks (Figure 4D,E) and LV rT3 was at or below the detection limit in all conditions. The tissue/plasma ratio of T4 was increased significantly after two weeks, whereas the tissue/plasma ratio of T3 did not change significantly (Figure 4F,G).

LV D3 activity most likely played a role in the low LV T3 levels as it was strongly induced at week one (Figure 4H), with the highest absolute activities similar to those previously reported for other models of pathological ventricular hypertrophy (see Discussion). Surprisingly, D3 activity returned to levels not significantly different from controls after two weeks.

**Figure 5:**
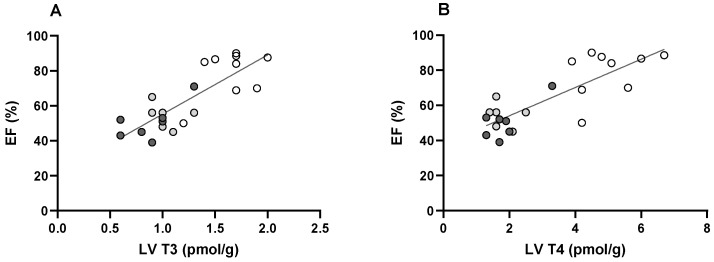
Ejection fraction as a function of LV tissue T3 content (**A**) and T4 content (**B**). Data are from Figure 3E (ejection fraction) and Figure 4C,D (LV T4 and T3). Regression lines: (**A**): R^2^ = 0.66, slope 33.8 (*p* < 0.0001); (**B**): R^2^ = 0.67, slope 8.1 (*p* < 0.0001). Symbols: CON: open; ISO + PE 1 week: light grey; ISO + PE 2 week: dark grey.

The possible relevance of cardiac thyroid hormone levels for LV function is indicated by the positive correlation of these levels and the ejection fraction (Figure 5A,B).

## 3. Discussion

The objective of this study was to provide a comprehensive analysis of the development of compensated and decompensated LV hypertrophy, including the determination of both plasma and LV tissue TH levels as well as D3 activity. Together these analyses would indicate firstly to what extent changes in LV TH levels may be the result of cardiac D3 activity and/or changes in systemic TH homeostasis, and secondly, whether these changes may play a role in the development of LV contractile dysfunction.

We used chronic adrenergic stimulation as a model because of the well-established induction of ventricular hypertrophy by isoproterenol (ISO) and phenylephrine (PE), and the relative ease of administration of these agonists. Earlier studies using doses of ISO and/or PE ranging from 0.3 to 100 mg/kg.d for up to 26 days showed the induction of LV remodeling to varying degrees [17,22,23,24,25,26,27,28,29,30]. All studies used osmotic minipumps to infuse the agonists, with the exception of the study by Ueta et al. [17] where daily injections of ISO were used. Based on the collective data, we chose infusion for one and two weeks of ISO and ISO + PE, at 30 mg/kg.d of either agonist, to induce compensated and decompensated hypertrophy, respectively.

We used the cD3KO-CS mouse line without inducing the expression of inactive cardiac D3 protein. Pilot studies had shown that MI in these mice resulted in LV remodeling and a level of D3 induction after two weeks similar to what we previously reported for wild type C57BL/6J mice [14]. The cD3KO-CS line will ultimately allow for the assessment of the relevance of D3 activity for pathological ventricular remodeling and heart failure [31].

The observed 25–40% increases in LV weight (Figure 1A and Figure 3A) are in line with the 15–50% increases reported previously [17,22,23,24,25,26,27,28,29,30]. The comparison of our echocardiography data is limited to studies used by Kudej et al. [24] and Matkovich et al. [28], who also used 30 mg ISO/kg.d. They showed a 10% increase in LVEDD after one week [24] and a 20% increase after one and two weeks [28]. The average LVEDD in our ISO study, which was used to calculate the LV end-diastolic volumes (Figure 1B), was increased by 30% after two weeks (CON: 3,0 ± 0.3 mm vs. ISO: 3.9 ± 0.5 mm, means ± SD, *n* = 8).

The LV dilation in the ISO-treated mice after two weeks was associated with a reduction of the ejection fraction (Figure 1E). However, cardiac output remained normal (Figure 1H) and hypertrophy was therefore compensatory, as was the case in the previous ISO studies [24,28]. The 25% lower LV T3 content after two weeks (Figure 2D) could play a role in the observed reduction of contractility at this time point. Reduced transcriptional regulation by T3 contributes to the shift in contractile protein isoforms from the fast myosin heavy chain α-isoform Myh6 to the slow β-isoform Myh7, as well as the down regulation of the expression of sarcoplasmic reticulum Ca^2+^-ATPase (SERCA2a) and changes in other cardiac genes [6,32]. Earlier we reported a 50% lower LV T3 content two weeks post MI, with a concomitant reduction in T3-dependent transcriptional activity [14]. The shift in Myh isoform expression in that study was associated with the induction of LV D3 activity to an average level of 0.6 fmol/mg.min. Clearly, the non-significant increase in D3 activity at two weeks to levels less than 0.04 fmol/mg.min (Figure 2G) cannot account for the lower LV T3 or T4 content. Yet, plasma TH levels were not reduced and Figure 3E,F suggest a different mechanism affecting LV TH levels.

Cardiac TH levels under normal conditions are thought to be determined by plasma levels with uptake mediated by monocarboxylate transporters MCT8 and MCT10 [33]. Since the local conversion of T4 to T3 has been shown to be negligible in rodent myocardium [34], a reduced uptake of TH is at present the most plausible explanation for the lower LV TH levels. However, the expression of cardiac MCT8 and MCT10 has so far only been examined at the mRNA level in diabetic cardiomyopathy, showing upregulation of MCT8 and downregulation of MCT10 [18].

Given the already substantial remodeling induced by ISO, it is not surprising that the additional hemodynamic load imposed by PE tipped the balance to decompensation (Figure 3). PE increases blood pressure, and consequently afterload, through elevation of systemic vascular resistance, without directly affecting cardiac contractility [26,35]. The additive effects of ISO and PE were shown in a study by Saadane et al. where heart weight increased by 13% after two weeks of ISO (30 mg/kg.d) and by 41%, when ISO was combined with PE (29 mg/kg.d) [26]. Whether this affected cardiac function was not assessed in that study.

The development of decompensation over the course of two weeks in the ISO + PE series is evident when comparing Figure 1 and Figure 3, with a lesser degree of hypertrophy and a greater reduction of fractional shortening than seen with ISO alone, already after one week. After two weeks, the drop in cardiac output, the myocardial atrophy and loss of body weight signal severe heart failure and associated cachexia. The marked changes in systemic TH homeostasis (Figure 4) are evidence of the excessive hemodynamic load induced by the combination of ISO and PE. The reduction of plasma TH levels is characteristic of various forms of acute or chronic stress on the system, such as surgery, starvation and severe illness, including heart failure [11,36,37].

In mouse and rat models of heart failure, both transiently and stably reduced circulating levels of T3 and/or T4 have been reported [12,13,14,16,17,38,39]. The decrease in T3 levels, and in severe illness also T4, while thyroid stimulating hormone (TSH) may remain normal, is known as the non-thyroidal illness syndrome (NTIS). The pathogenesis is multifactorial and may include alteration of the set-point of the hypothalamus–pituitary–thyroid (HPT) axis or changes in peripheral TH metabolism [11]. The latter has for instance been shown in studies in the livers of critically ill patients. Reduced circulating levels of T3 and T4 were associated with the reduced activation of TH by deiodinase type I (D1), and increased inactivation of TH by D3 [40]. Liver D1 also plays a key role in the breakdown of rT3 and, consequently, the reduced D1 activity in these patients correlated with increased circulating rT3 levels [40,41]. However, normal or reduced levels of rT3 may also occur in NTIS [11]. The current study is the first to examine plasma rT3 levels in a rodent model of heart failure. The parallel decrease in T4 and rT3 (Figure 4) suggests that an altered set-point of the HPT axis is responsible for the observed drop in TH levels, rather than a change in peripheral metabolism.

The high levels of LV D3 activity seen at week one, when plasma T3 levels were not yet fully reduced, may have contributed to the low LV T3 content at this time point (Figure 4). Ueta et al. [17] also reported a significant increase in LV D3 activity after 10 daily injections of 100 mg ISO/kg, but later time points were not included in that study. Our data at week two clearly indicate that the induction of D3 activity is transient and that plasma TH levels most likely are the determining factor in setting tissue TH levels. The transient nature of the induction of D3 activity most likely also accounts for the observed variation in activities at week two.

As pointed out above, LV function is in part determined by the T3-dependent transcriptional regulation of several key proteins involved in contraction and relaxation. Although the ejection fraction is determined by a number of factors, the strong positive correlation shown for LV T3 in Figure 5 indicates the relevance of tissue T3 for LV function. T4 is considerably less potent than T3 in terms of transcriptional regulation, and the conversion of T4 to T3 by deiodinase type 2 (D2) is negligible to absent in the rat and mouse heart [12,34,42]. However, at the tissue levels we report here, it cannot be excluded that T4 plays a role as well.

Summarizing, compensated LV hypertrophy induced by chronic adrenergic stimulation for two weeks showed LV dilation with reduced ejection fraction, but normal stroke volume and cardiac output. Normal plasma TH levels and reduced LV tissue levels suggest diminished uptake of TH since LV D3 activity was not different from the control. In contrast, decompensated LV hypertrophy showed LV dilation with a greater reduction of the ejection fraction, reduced stroke volume and cardiac output, and loss of body weight. The substantial drop in plasma TH levels, which is not the result of peripheral metabolism, can account for the similar drop in LV tissue TH levels, but a transient induction of high LV D3 activity may have contributed to the low tissue TH levels after one week.

Unlike other models of pathological ventricular remodeling in mice and rats, the present data do not support a role for cardiac D3 activity in hypertrophy induced by adrenergic stimulation. However, the unexpectedly dynamic expression of D3 activity hints at a novel mechanism of regulation in the stressed heart. In addition, the marked and divergent changes in circulating and cardiac TH levels make these models well suited to examine the role of TH in cardiac dysfunction in heart disease as well as other severe illnesses.

## 4. Materials and Methods

### 4.1. Generation of the cD3KO-CS Strain

The D3KO strain [43] was used to generate the cardiac-specific, conditional D3 knock-out line (cD3KO-CS). In the D3KO strain, the selenocysteine insertion sequence (SECIS) in the 3′-UTR of the Dio3 gene is flanked by LoxP sites (Dio3^fl/fl^). The SECIS element is essential for the insertion of selenocysteine in the catalytic center of D3 [44] and the excision of the element results in the expression of a truncated and inactive D3 protein [43]. By crossing this strain with the C57BL6/J-congenic α-MHC-MerCreMer strain (Jackson Laboratory, stock number 005657), the C57BL6/J-Dio3^fl/fl^MerCreMer^+/-^ (cD3KO-CS) strain was obtained, in which tamoxifen activates Cre only in cardiomyocytes. All mice used in this study were homozygous for floxed Dio3 and heterozygous for α-MHC-MerCreMer. Tamoxifen treatment was not used in this study, and the expressed D3 protein is therefore enzymatically active.

### 4.2. Implantation of Osmotic Minipumps

A total of 51 mice of either sex, aged 12 weeks, were randomly assigned to the CON (10), ISO (16) and ISO + PE (15) groups. Osmotic minipumps (Alzet) model 1007D and model 1002 were used for 7-day and 14-day delivery, respectively. Following the manufacturer’s instructions, the pumps were filled with distilled sterile water containing 0.1% ascorbic acid, without addition (CON), with isoproterenol (ISO) or with isoproterenol and phenylephrine (ISO + PE). Concentrations were adjusted to animal weight to deliver 30 mg/kg.d of each agonist at a rate of 0.5 µL/h (7 days) or 0.25 µL/h (14 days). The analgesic buprenorphine (0.1 mg/kg) was injected 30 min prior to surgery. Pumps were placed in the intrascapular tissue under general anesthesia (2.5% isoflurane). Animals were housed with more than one per cage with free access to food and water.

### 4.3. Echocardiography

Mice were ventilated with 2.5% isoflurane and body temperature was maintained between 36.8 and 37.2 C [14]. Short axis and parasternal axis echocardiographic images were acquired and analyzed using a VisualSonics VeVo 2100 Imaging System (FUJIFILM VisualSonics Inc.) equipped with an MS-550D22-55 MHz transducer [45] to yield heart rate; LV diastolic and systolic anterior and posterior wall thickness; LV end-diastolic diameter (LVEDD); LV end-systolic diameter (LVESD); and LVED and LVES volumes.

LV hypertrophy was considered compensatory when cardiac output was maintained, and mice showed normal growth. A reduction of cardiac output combined with signs of distress and weight loss was considered to indicate decompensation.

### 4.4. Tissue Collection

Immediately following the acquisition of echocardiographic images, the chest was opened, blood was collected, and the heart was excised and rinsed. RV and LV + septum were dissected, weighed and frozen in liquid nitrogen. Both tibiae were dissected, and the average length was determined to normalize ventricle weights.

### 4.5. Determination of TH Levels in LV Tissue and Plasma

LV tissue levels of T4 and T3 were determined in 20–30 mg samples, as previously described [46]. Following extraction, the iodothyronines were separated and quantified using reversed phase chromatography followed by tandem mass spectrometry (LC-MS/MS) on an Acquity UPLC-Xevo TQ-S system (Waters, Milford, MA, USA). Plasma levels of T4, T3 and rT3 were determined using a recently published LC-MS/MS method to quantify TH and its metabolites [47]. Samples (250–500 μL) were deproteinized and TH and its metabolites were extracted, evaporated to dryness, and reconstituted. Samples were injected onto a Waters Acquity UPLC column and TH and its metabolites were measured with a Sciex QTRAP 6500+.

### 4.6. Determination of D3 Activity

D3 activity was determined in homogenates of 20–30 mg LV tissue by analysis of the conversion of ^125^I-labeled T3 to ^125^I-labeled T2, as previously described [48]. Samples were incubated at 37 °C for 1 h and metabolites were separated on a Waters HPLC system (Model 600E pump, Model 7171 WISP autosampler). The activity of the eluate was determined using a Radiomatic 150 TR flow scintillation analyzer (Perkin Elmer).

### 4.7. Statistical Analyses

The experiments were designed to determine the effect of agonist treatment on the presented parameters. Unless otherwise indicated, all data were analyzed by two-way ANOVA followed by an unprotected Fisher’s least statistical difference (LSD) test using GraphPad Prism 9 (GraphPad Software), with *p* < 0.05 considered significant.

## Data Availability

The data presented in this paper are available on request from the corresponding author. Data is not publicly available due to privacy or ethical restrictions
